# Frailty: A cost incurred by reproduction?

**DOI:** 10.1038/s41598-020-67009-2

**Published:** 2020-06-23

**Authors:** E. H. Gordon, N. M. Peel, M. D. Chatfield, I. A. Lang, R. E. Hubbard

**Affiliations:** 1 Centre for Health Services Research, The University of Queensland, Brisbane, Australia; 2 The University of Exeter, Exeter, United Kingdom

**Keywords:** Ageing, Epidemiology

## Abstract

Evolutionary theories of senescence, such as the ‘disposable soma’ theory, propose that natural selection trades late survival for early fecundity. ‘Frailty’, a multidimensional measure of health status, may help to better define the long-term consequences of reproduction. We examined the relationship between parity and later life frailty (as measured by the Frailty Index) in a sample of 3,534 adults aged 65 years and older who participated in the English Longitudinal Study of Ageing. We found that the most parous adults were the most frail and that the parity-frailty relationship was similar for both sexes. Whilst this study provided some evidence for a ‘parity-frailty trade-off’, there was little support for our hypothesis that the physiological costs of childbearing influence later life frailty. Rather, behavioural and social factors associated with rearing many children may have contributed to the development of frailty in both sexes.

## Introduction

The ‘disposable soma’ theory of ageing proposes that investing in reproduction, at the cost of somatic maintenance, leads to senescence^1^. Evidence for this evolutionary theory was first described in studies of the fruitfly, *Drosophila* spp., where selecting for lifespan was associated with changes in fecundity^1^. Studies using models with impaired reproduction due, for example, to neutering have yielded data supporting a reproduction-longevity trade-off in other species^2,3^.

In humans, the disposable soma theory predicts that those with more children will have shorter lives. In their seminal paper, Westendorp and Kirkwood^4^ used a historical dataset from the British aristocracy to demonstrate that females with the longest life span had fewer children relative to the whole sample. Indeed, almost 50% of females who lived to 80 years and over were childless. A similar relationship between parity and longevity was found in males. They concluded that their findings demonstrated a reproduction-longevity trade-off in humans and that their results were consistent with those derived from studies of non-human species. Westendorp and Kirkwood’s study^4^ has been criticized in the literature, particularly with regards to the quality of the data, the approach to statistical analysis and the authors’ conclusions^5^. Even so, its publication reignited interest in the reproduction-longevity relationship.

Despite a sustained research effort and strong theoretical expectations, evidence to support a reproduction-longevity trade-off in humans is not strong. Studies of historical and contemporary cohorts have not found a consistent association between parity and longevity – no association, as well as positive and negative associations, have all been reported^6–9^. A ‘J-shaped’ relationship between parity and mortality risk has also been described by a number of studies^10–12^. This non-linear association was found in a recent systematic review and meta-analysis, with the greatest reduction in all-cause mortality risk detected in males and females with three or four children^13^. This relationship suggests that there are ‘health advantages’ associated with moderate parity over nulliparity and high parity. Alternatively, moderately parous individuals may differ from nulliparous and highly parous individuals with respect to other important, health-related characteristics, such as socio-economic status.

Many, but not all, studies exploring the relationships between reproduction and longevity have presented sex-stratified results. Although most have shown that the *direction* of the associations is the same for both sexes, the *effect size* has been shown to differ. For example, in some cases childlessness was found to be more disadvantageous (in terms of survival) in females than in males^7,11,12^ and in others, high parity was found to be more disadvantageous for males than females^11,12^. The pathways linking reproduction with later life health in males and females remain unclear. However, the disposable soma theory continues to be cited in the literature^13,14^. Certainly, females invest significant physiological resources into pregnancy, childbirth and lactation and this may be at the expense of somatic maintenance; however, it is less clear how a trade-off would transpire in males. Furthermore, the disposable soma theory fails to account for sex differences in longevity, whereby females consistently live longer than males^15^. Since a biological explanation for a relationship between reproduction and longevity (or other health outcomes) in males is unlikely, non-biological factors must play a role. That is, childrearing must also impact ageing trajectories.

Most studies to date have tested evolutionary theories of senescence by focusing on the relationship between parity and survival (usually measured in terms of longevity). However, it is possible that survival is too crude a measure of senescence and, as a result, the ‘real’ cost incurred by reproduction has not been elucidated. Whilst studies have examined other health outcomes, such as physical, functional and cognitive impairment, self-rated health and limiting long-term illnesses in older males and females^9,16–24^, findings have not been consistent. It is likely that between-study differences in defining and measuring multiple health outcomes is a key factor contributing to discordant results. Furthermore, examining the relationships between parity and individual domains of health may not be the best methodology to address the hypothesis because impairment profiles vary significantly in the older adult population and measures of individual domains do not capture all adults with poor health. ‘Frailty’, on the other hand, is a multidimensional measure of health status that may help to better define the long-term consequences (whether they be harms or benefits) of human reproduction.

Frailty has been defined as a state of increased vulnerability to stressors that is associated with adverse health outcomes, including mortality, disability and institutionalization^25^. In a frail individual, accumulated damage to various physiological systems leads to a reduced ability to compensate for disruptions to homeostasis. Frailty, therefore, signifies senescence. The cumulative deficit model of frailty, represented by a continuous variable called the Frailty Index (FI), proposes that the more problems (or ‘deficits’) an individual has acquired, the more likely they are to be frail^26^. The FI, which ranges from zero to one, predicts adverse outcomes in a dose-dependent manner^27–29^ and its validity has been confirmed by multiple studies conducted in a variety of settings using cohorts from different cultural backgrounds^30–32^.

The aims of this study were to examine the cross-sectional relationship between parity and later life frailty (represented by the FI) and to explore whether this relationship is influenced by sex. Data from the English Longitudinal Study of Ageing (ELSA) were used to test two key hypotheses: firstly, that higher parity is associated with greater frailty, indicating a ‘parity-frailty trade-off’ and secondly, that sex differences in frailty are greater at higher parities than at lower parities due to sex differences in the physiological costs of childbearing.

## Results

### Sample characteristics

Three thousand five hundred thirty-four community-dwelling adults aged 65 years and older (55.9% female) were included in the analysis. Of this sample, five (0.1%) resided in aged care facilities. The FI derived from the ELSA dataset (see Methods) had a skewed distribution with a mean score of 0.16 (standard deviation (SD) = 0.12) and a median score of 0.14 (interquartile range (IQR) = 0.08–0.24). The maximum FI was 0.66 and the 99^th^ percentile was 0.53.

The sample characteristics stratified by sex are presented in Table 1. The proportion of participants aged 85 years and older was higher in females than males (Χ^2^ = 15.27, df = 4, p = 0.004) and the mean FI was higher in females than in males of the same age (mean difference = 0.034, 95% CI = 0.026–0.041, p < 0.001). With regards to reproductive characteristics, the distribution of parity was similar for both sexes, with two children being the most common parity in this sample (Χ^2^ = 5.77, df=6, p = 0.45).

**Table 1:** Characteristics of sample participants.

	Female N = 1974	Male N = 1560
**Age** N (%)		
65–69	545 (27.6)	455 (29.2)
70–74	517 (26.2)	453 (29.0)
75–79	432 (21.9)	324 (20.8)
80–84	267 (13.5)	214 (13.7)
85+	213 (10.8)	114 (7.3)
**Frailty Index** mean (SD)	0.18 (0.13)	0.14 (0.11)
**Parity** N (%)		
0	274 (13.9)	191 (12.2)
1	278 (14.1)	221 (14.2)
2	687 (34.8)	594 (38.1)
3	436 (22.1)	322 (20.6)
4	189 (9.6)	140 (9.0)
5	65 (3.3)	56 (3.6)
6+	45 (2.3)	36 (2.3)

### The relationship between parity and frailty

In a main effects model including age, sex and parity, all variables had a statistically significant effect on frailty (age: F(4,3522) = 103.09, p < 0.001; sex: F(1,3522) = 76.90, p < 0.001; parity: F(6,3522) = 3.89, p < 0.001).

**Figure 1:**
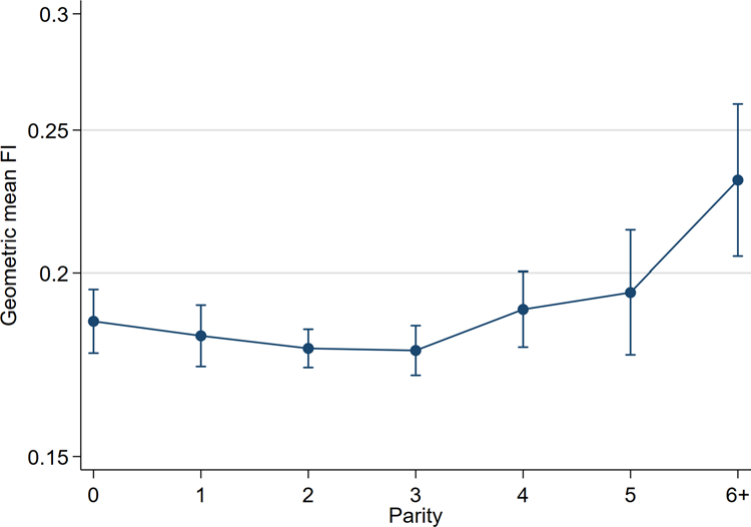
Graph of geometric mean Frailty Index (FI) with 95% confidence intervals for each parity category (using adjusted predictions for age and sex).

The relationship between parity and frailty (adjusted for age and sex) is presented in Table 2 and Fig. 1. Those with high parity (6 + children) had significantly higher frailty than those without children, whereas those with one to five children had similar frailty to those without children. Even so, visual inspection of the relationship between parity and frailty suggests a trend towards lower frailty in those with two to three children.

**Table 2:** Relationships between categorical independent variables and the Frailty Index (FI) in the main effects model.

Independent Variable		GMR	95% CI	p-value
Parity	0	Ref		
	1	0.98	0.91–1.05	0.530
	2	0.96	0.91–1.02	0.162
	3	0.96	0.90–1.02	0.166
	4	1.02	0.94–1.10	0.631
	5	1.05	0.94–1.17	0.413
	6+	1.25	1.10–1.42	0.001
Sex	Male	Ref		
	Female	1.18	1.13–1.22	<0.001
Age	65–69	Ref		
	70–74	1.14	1.08–1.19	<0.001
	75–79	1.31	1.25–1.38	<0.001
	80–84	1.48	1.40–1.58	<0.001
	85+	1.86	1.74–1.99	<0.001

Note.

GMR: geometric mean ratio; CI: confidence interval; Ref: reference group.

**Figure 2:**
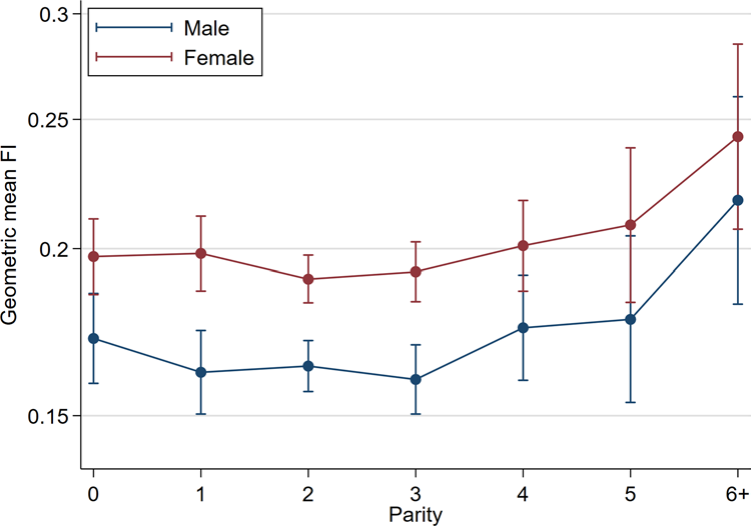
Graph of geometric mean Frailty Index (FI) with 95% confidence intervals for each sex and parity (adjusted for age).

**Figure 3:**
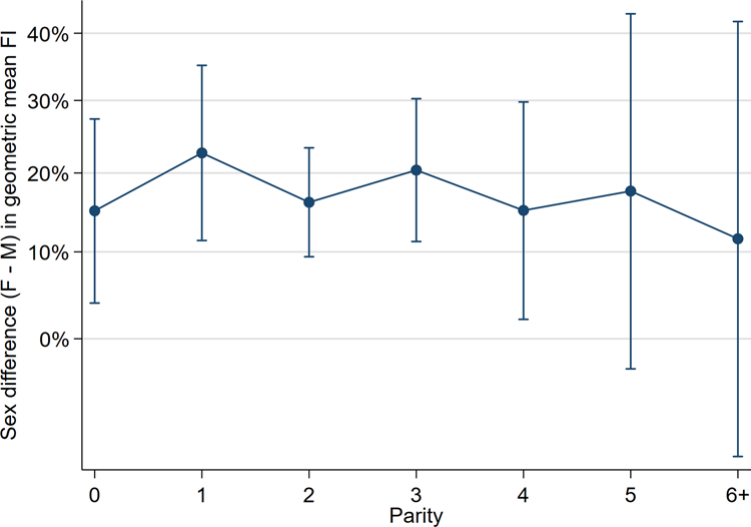
Graph of sex differences in geometric mean Frailty Index (FI) with 95% confidence intervals for each parity (adjusted for age).

The geometric mean FI was higher in females than males in all parity categories (Fig. 2). The *relative* sex difference in FI (adjusted for parity and age) was 18% (95% CI = 13–22, p < 0.001) (Table 2) (Fig. 3). The interaction between sex and parity was not significant (F(1,3521) = 0.03, p = 0.867).

A second main effects model assessed the confounding effects of education on the parity-frailty relationship. The education variables had statistically significant effects on frailty. However, there was a negligible effect on the relationship between parity and frailty (see Supplementary Information).

## Discussion

In this study, we examined the relationship between reproduction and later life health in males and females using the cumulative deficit model of frailty (the FI). We found that the most parous adults were the most frail, providing weak evidence for a ‘parity-frailty trade-off’. The relationship between parity and frailty was similar for both sexes and the relative difference between male and female FIs was fairly consistent across all parity categories, including nulliparity. Overall, the results suggest that behavioral and social factors associated with rearing many children may be more relevant to the parity-frailty relationship than the physiological burden of childbearing.

The results of this study cannot be corroborated by the frailty literature, which to date has not explored the relationship between parity and frailty. However, in 2015, Grundy and Read^22^ examined ‘allostatic load’ in relation to fertility history and later life health. Allostatic load, defined as an indicator of *“multisystem physical dysregulation resulting from cumulative effects of responding to multiple stressors”* (p.113), is calculated from a set of biomarkers^22^. It is conceptually similar to the accumulative deficit model of frailty^33^. Despite notable differences in the categorization of parity and reference groups, their results align with those of the current study; they found that, high parity (which they defined as four or more children) was associated with higher (worse) allostatic load than moderate parity (two children) in both sexes^22^. Whilst they did not report whether males and females in their study differed with respect to the degree of allostatic load^22^, the direction and magnitude of the associations appeared to be similar.

A dose-response relationship between parity and frailty, indicating a ‘parity-frailty trade-off’, was anticipated in this study. High parity (6 or more children) was associated with greater frailty, relative to nulliparity, in this sample; however, there were no significant differences between the FIs of nulliparous adults and the FIs of adults with up to five children. In the research literature, a ‘J-shaped’ relationship, characterized by a decrease in mortality risk with an initial increase in parity, has been reported and several potential mechanisms have been discussed. For example, parenthood appears to have a positive impact on health-related behaviours (such as alcohol use), social participation and social support in both sexes^22^. Reproduction may be beneficial to the later life health of males due to the associated positive effects of marriage and partnership^34^ and in females, pregnancy may exert a protective, biological effect. For example, multiparity has been associated with reduced risk of reproductive cancer^35^ and in a study of cardiovascular disease-related mortality, moderate parity (defined as four children) appeared to have a protective effect, possibly mediated by enhanced endothelial function persisting postpartum^36^. However, the apparent advantageous effects of parity may in fact be ‘selection effects’; that is, adults with poor health in childhood and their reproductive years may be selected into childlessness or low parity^37^. Poor health in early life may also dictate health in later life. Indeed, a recent study identified an association between extremely early health measures, such as small birth weight, and frailty in older Finnish men and women^38^.

On visual inspection of the data there appeared to be a trend towards lower frailty in those with two or three children (compared with those with no children) and a trend towards higher frailty in those with four or five children (compared with those with two or three children). Thus, it is possible that there is a J-shaped relationship between parity and frailty, but it is too subtle to capture with a study of this sample size. Indeed, in a meta-analysis of parity and mortality studies including over 2.8 million participants, absolute risk reductions of 0.02 to 0.04 were found to be statistically significant^13^. An alternative view, is that later life frailty is only associated with high parity. This would indicate that there are important biological or sociodemographic factors unique to that group of individuals that require further exploration.

The idea that high parity, characterized by repeated pregnancies, childbirths and periods of lactation, leads to an accumulation of deficits and contributes to later life frailty in females seems logical. However, the pathways linking childbearing to frailty require consideration. According to the disposable soma theory, an investment of resources into childbearing results in suboptimal somatic maintenance; however, the mechanism(s) by which this occurs, the nature of the damage sustained and the body systems affected are not well understood. An alternative (or additional) hypothesis is that repeated exposure to the physiological changes of childbearing may contribute directly to the pathophysiology of frailty. For example, there is evidence that pregnancy is accompanied by an inflammatory milieu^39,40^ and, based on what is known about the relationship between inflammation and frailty, it is possible that this contributes to the development of frailty in multiparous females. The energetic and nutritional requirements of childbearing may be particularly costly^41^. For well-nourished females in developed countries, increasing caloric intake or decreasing physical demands may be sufficient to counteract physiological consequences of childbearing^41^. However, if resources are limited (due to poor diet, need for ongoing intense physical activity or intercurrent illness), repeated childbearing may lead to a state of ‘maternal depletion’, drawing energy away from other body systems and increasing the risk of frailty. High parity may also contribute to the development of frailty via increased rates of metabolic disease and cardiovascular disease. In a recent meta-analysis^39^ there was a linear, dose-response relationship between parity and type two diabetes, but females with three or more children were at a significantly elevated risk. Similarly, another meta-analysis demonstrated that females with more than four children faced a significantly higher risk of cardiovascular disease-related mortality^36^. There are several mechanisms that may underpin increased rates of these diseases in high parity. For example, pregnancy induces vascular changes and a pronounced state of insulin resistance and, as a result, repeated pregnancies may result in long-term alterations to vasculature and glucose homeostasis^36,39,40^. Furthermore, redistribution of adipose tissue and recurrent periods of gestational weight gain may predispose to postpartum obesity^42^. The dose-response relationship between parity and urinary incontinence (attributed to pelvic floor injuries sustained during pregnancy and childbirth)^43^ may also contribute to the development of frailty in multiparous females.

In this context, we expected to find that sex differences in frailty (wherein females were more frail than males) were greater at higher parities than at lower parities. However, the analyses did not demonstrate an interaction between sex and parity in relation to later life frailty: the relative difference in female and male FIs was approximately 18% across all parity categories and the relationship between parity and frailty was similar in both sexes. That is, in both sexes, those with high parity were more frail than their nulliparous counterparts. Again, it is possible that the sample size was not sufficient to demonstrate an interaction between sex and parity (in relation to frailty). Indeed, high parity was not very prevalent in this sample (i.e., only 2.3% of males and females had six or more offspring) and modest sample sizes contributed to reduced accuracy of the estimates in the higher parity categories (i.e., 4, 5 and 6+ parity categories). The finding that females were more frail than males in this sample of community-dwelling adults is consistent with the frailty literature^44^ and FI-age-sex analyses (see Supplementary Information) showed that the arithmetic mean FI of females was higher than males in all age groups and the relative difference in FIs was approximately 18% (see Supplementary Figures 4 and 5). Overall, the findings do not support the hypothesis that the cumulative, damaging effects of childbearing contribute to later life frailty. Rather, the results suggest that behavioural and social factors associated with rearing many children may be relevant to the development of later life frailty in both sexes.

A parity-frailty trade-off may manifest in older males and females with high parity due to economic strain, disruption of occupational attainment and psychological stress^22,45^. In addition, high parity may negatively influence lifestyle habits such as dietary choices and physical activity in both sexes^22^. These behavioural factors increase the risk of obesity and its metabolic complications, which in turn, increase the risk of frailty. These explanations, however, rest on the assumption that biological parents participate in the rearing of their offspring. An alternative theory is that selection effects confound the relationship between high parity and frailty. For example, lower levels of education level are associated with particular reproductive characteristics, such as early parenthood and higher overall parity^45^, as well as later life frailty^31^. However, in this study, education was not found to have a significant impact on the parity-frailty relationship.

To our knowledge, this study was the first to use frailty to test evolutionary theory linking reproduction with ageing. In this study, frailty was conceptualized as a measure of senescence and the results make a novel contribution to a vast body of work regarding reproduction and the life history of the human species. It is important, however, to consider the study findings in the context of its limitations. Firstly, the cross-sectional nature of the data precludes the inference of causality. Furthermore, this study only included age and educational level as potential confounders in its analyses. There are other biological, behavioural, socioeconomic and environmental factors associated with later life frailty^46^ and many of these factors may also be associated with reproduction. Consequently, there are likely to be residual confounding effects. This study used biological sex to explore whether the physiological effects of childbearing mediates the association between parity and frailty. However, health-related behaviours, economic burden and psychosocial strain may also mediate the parity-frailty relationship and warrant further exploration.

The results of this study may also have been impacted by characteristics of the sample. For example, comprehensive reproductive data were available for a subset of Wave 3 participants only and these participants were less likely to have poor self-rated health and poor socioeconomic status, were less likely to require proxy interviews and were, on average, younger. Furthermore, the distribution of parity was heavily skewed to the right and whilst this is typical of contemporary populations from more developed countries^41^, this may have impacted the magnitude of the signal in this study. It is also important to consider the risk of measurement bias in studies of reproductive histories. In particular, under-reporting of non-marital births may have led to an underestimation of parity in both sexes. Also, this study only included adults aged over 65 years and, as a result, survivor bias may have contributed to an underestimation of the relationship between parity and frailty. Whilst a prospective cohort study may minimize these issues, the long duration of follow-up may limit the feasibility of such an investigation.

In conclusion, this study provided weak evidence for a trade-off between high parity and later life frailty. Stratifying the results by sex suggested that a parity-frailty relationship is likely to be primarily influenced by psychological, behavioural and socioeconomic factors associated with childrearing, rather than the physiological effects of childbearing. The findings of this study inspire further exploration of reproductive history and biopsychosocial factors as they relate to the development of frailty in ageing males and females.

## Methods

### Study sample

The English Longitudinal Study of Ageing (ELSA) is a nationally representative panel study of adults aged 50 and over (and their partners) living in private households in England. Participants were recruited from households involved in the Health Survey for England (HSE) in 1998, 1999 and 2001.

The original sample consisted of 12 099 adults aged between 50 and 100 years. Comparison of the sociodemographic characteristics of the sample against national census data indicated that the sample was representative of the English population^47^.

Biannual study waves, the first of which occurred in 2002, included face-to-face interviews and self-completion questionnaires with both members of couples in age-eligible households. At each wave, ELSA collected data regarding several topics, including household and individual demographics, physical and psychological health, work and pensions, income and assets, housing, social participation and cognition. In Wave 3, participants who completed the interview in person were invited to complete a ‘Life History’ interview. This interview collected data regarding the participant’s childhood and early adult life. Topics included reproduction, past and current relationships, employment, childhood illnesses and parental relationships.

The sample for this study were aged 65 years and older, completed the Life History interview and were interviewed in person (not via proxy). This age group was chosen to maximise the prevalence of frailty and to focus on the relationship between reproduction and frailty in older age (as opposed to middle-age).

### Frailty index

An FI constructed from 40 variables was used for secondary analysis in this study (see Supplementary Information). Variables were taken from ‘core topics’ covered in each ELSA wave. Variables were required to satisfy five established criteria: they must be associated with health status; they must reflect a range of systems; their prevalence must increase with age; they must not saturate too early; and, they must be the same across iterations (if an FI is to be used serially on the same sample)^48^. Binary variables were coded using established convention (i.e., ‘0’ indicated the absence of the deficit and ‘1’ indicated the presence of a deficit). Ordinal and continuous variables were coded based on clinical judgment and distribution of data. For example, a deficit for memory impairment was defined as a score within the lowest quartile of the sample. Each individual’s deficits were summed and then divided by the total number of potential deficits (e.g., if an individual has 10 deficits from a total of 40 potential deficits, they have an FI of 0.25). For those with missing items, the denominator was reduced accordingly. To be included in the sample, participants were required to have an FI denominator equal to or greater than 30, since FIs with a denominator of at least 30 have been shown to be sufficiently accurate^48^.

### Parity

Parity was a count of children that were born alive (including children from whom participants were estranged and children who had died).

### Statistical analysis

Age data were presented in categories because the exact age of participants aged over 90 years was not provided in the dataset. Based on the distribution of the data, parities of six or more children were combined into a single category (6 + ).

Sample characteristics, stratified by sex, were described using means and standard deviations (for continuous data) and percentages (for categorical data). Differences between the sexes were assessed using independent t-tests and chi-square tests for continuous and categorical variables, respectively.

Several models were used to evaluate the relationship between parity, frailty and sex. Following visual inspection of the relationship between age and parity, age group was included as a confounding variable. The transformed FI was the dependent variable in all analyses:

$$\text{log}\,\text{FI}\,=\,\text{log}(\text{FI}\,+\,0.05)$$

In order to present the transformed data on the original scale (i.e., an FI ranging from 0–1), ‘geometric means’ were calculated as follows:

$$\text{Geometric}\,\text{mean}\,=\,\text{exp}(\text{mean}(\text{log}(\text{FI}\,+\,0.05)))$$

To compare the geometric mean FIs of males and females, geometric mean ratios (GMR) were calculated. In this instance, male sex was the reference category.

**Table:** 

Model	Independent Variables	Dependent Variable
Main effects	Age group, sex and parity	logFI
Interaction	Main effects and the interaction between sex and parity (continuous)	logFI
Interaction	Main effects and the interaction between sex and parity (categorical)	logFI

A graphical representation of the relationship between parity and frailty was derived using adjusted predictions for age group and sex from the main effects model. To compare the geometric mean FIs of different parity categories, GMRs were calculated with nulliparity as the reference category.

Analyses were repeated with education variables included as potential confounders. The two education variables were qualification level (i.e., <O-Level, O-Level or A-Level,> A-Level) and age (in years) that the participant completed full-time education.

**Table:** 

Model	Independent Variables	Dependent Variable
Main effects	Age group, sex, parity, qualification level and age completed education	logFI
Interaction	Main effects and the interaction between sex and parity (continuous)	logFI
Interaction	Main effects and the interaction between sex and parity (categorical)	logFI

The level of statistical significance was set to p < 0.05. STATA (release 15) and SPSS Statistical Software (version 25.0) were used for statistical analysis^49,50^.

### Ethical clearance and data access

Ethical approvals for all ELSA waves were granted according to the ethical approval system in operation at the time. For example, ethical approval for Wave 3 was granted from the National Hospital for Neurology and Neurosurgery and Institute of Neurology Joint Research Ethics Committee. All participants provided informed consent and all methods were carried out in accordance with relevant guidelines and regulations. All data and documentation (with the exception of sensitive data) are deposited in the Economic and Social Data Service archive. Data are completely anonymized. Access to ELSA data for secondary analysis in this study was approved on application to the UK Data Service.

## Supplementary information

Supplementary Information.
