# Supplementary material for: Frailty: A cost incurred by reproduction?
Source: Sci Rep. 2020 Jun 23;10:10139. doi: 10.1038/s41598-020-67009-2 (PMC7311439; doi:10.1038/s41598-020-67009-2)
Supplement: Supplementary file 1 — Supplementary Information. [file 41598_2020_67009_MOESM1_ESM.docx]

**Supplementary Information**

**Frailty: A cost incurred by reproduction?**

EH Gordon *^a^, NM Peel ^a^, MD Chatfield ^a^, IA Lang ^b^, RE Hubbard ^a^

^*^ Corresponding Author (email: e.gordon@uq.edu.au)

^a^ Centre for Health Services Research, The University of Queensland, Brisbane Australia

^b^ The University of Exeter, Exeter, United Kingdom

**FI-Age-Sex-Education Analyses**

**Results**

|  | | **Model 1** | | **Model 2** | |
| --- | --- | --- | --- | --- | --- |
| **Independent Variable** | | **GMR** | **p-value** | **GMR** | **p-value** |
| Parity | 0 | Ref |  | Ref |  |
|  | 1 | 0.98 | 0.53 | 0.96 | 0.27 |
|  | 2 | 0.96 | 0.16 | 0.96 | 0.12 |
|  | 3 | 0.96 | 0.17 | 0.95 | 0.10 |
|  | 4 | 1.02 | 0.63 | 1.02 | 0.68 |
|  | 5 | 1.05 | 0.41 | 1.04 | 0.47 |
|  | 6+ | 1.25 | 0.001 | 1.19 | <0.01 |
| Sex | Male | Ref |  | Ref |  |
|  | Female | 1.18 | <0.001 | 1.15 | <0.001 |
| Age | 65-69 | Ref |  | Ref |  |
|  | 70-74 | 1.14 | <0.001 | 1.11 | <0.001 |
|  | 75-79 | 1.31 | <0.001 | 1.25 | <0.001 |
|  | 80-84 | 1.48 | <0.001 | 1.42 | <0.001 |
|  | 85+ | 1.86 | <0.001 | 1.77 | <0.001 |
| Age finished full-time education | Continuous variable |  |  | 0.99 | <0.01 |
| Qualification Level | < O-level |  |  | Ref |  |
|  | O or A-level |  |  | 0.87 | <0.001 |
|  | > A-level |  |  | 0.83 | <0.001 |

**Table 2** Relationships between categorical independent variables and the Frailty Index (FI) in main effects models with (Model 2) and without (Model 1) education variables.

Note.

GMR: geometric mean ratio; Ref: reference group

**FI-Age-Sex Analyses**

**Methods**

Several models were used to evaluate the relationship between sex, age and FI. First, a*bsolute* sex differences were assessed using untransformed FI values.

| **Model** | **Independent Variables** | **Dependent Variable** |
| --- | --- | --- |
| 1: Main effects | Age group and sex | FI |
| 2a: Interaction | Age group, sex and the interaction between age group (continuous) and sex | FI |
| 2b: Interaction | Age group, sex and the interaction between age group (categorical) and sex | FI |

Second, *relative* sex differences were assessed using transformed FI values:

logFI = log(FI+0.05)

In order to present the transformed data on the original scale (i.e., an FI ranging from 0-1), ‘geometric means’ were calculated as follows:

Geometric mean = exp(mean(log(FI+0.05)))

To compare the geometric mean FIs of males and females, geometric mean ratios (GMR) were calculated. In this instance, male sex was the reference category.

| **Model** | **Independent Variables** | **Dependent Variable** |
| --- | --- | --- |
| 3: Main effects | Age group and sex | logFI |
| 4a: Interaction | Age group, sex and the interaction between age group (continuous) and sex | logFI |
| 4b: Interaction | Age group, sex and the interaction between age group (categorical) and sex | logFI |

**Results**

Figure 1 demonstrates the skewed distribution of FI for each sex in each age group. The median FI is higher in females than males in all age groups. The mean FI in females was higher than males in all age groups and mean FI increased with age in both sexes (Figure 2). The main effects of age and sex on FI were significant (F(4,3528)=97.31, p<0.001 and F(1,3528)=71.23, p<0.001, respectively). There was a significant interaction between age and sex (F(1,3527)=4.18, p=0.041), indicating that the sex difference in FI varied linearly with age group (Figure 3). The smallest a*bsolute* sex difference in FI was 0.024 (70-74 years) and the largest was 0.049 (80-84 years).


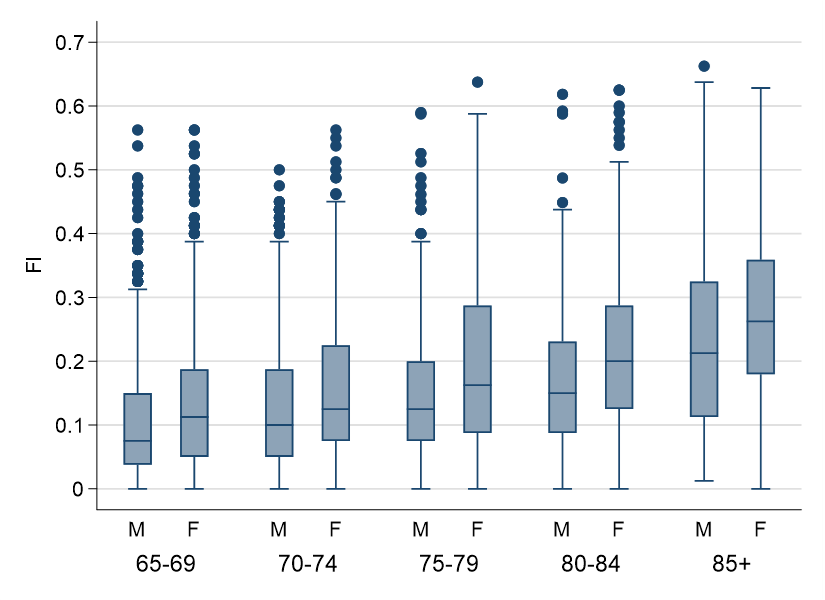


**Figure 1** Boxplots demonstrating median Frailty Index (FI; with inter-quartile ranges) for each sex and age group.


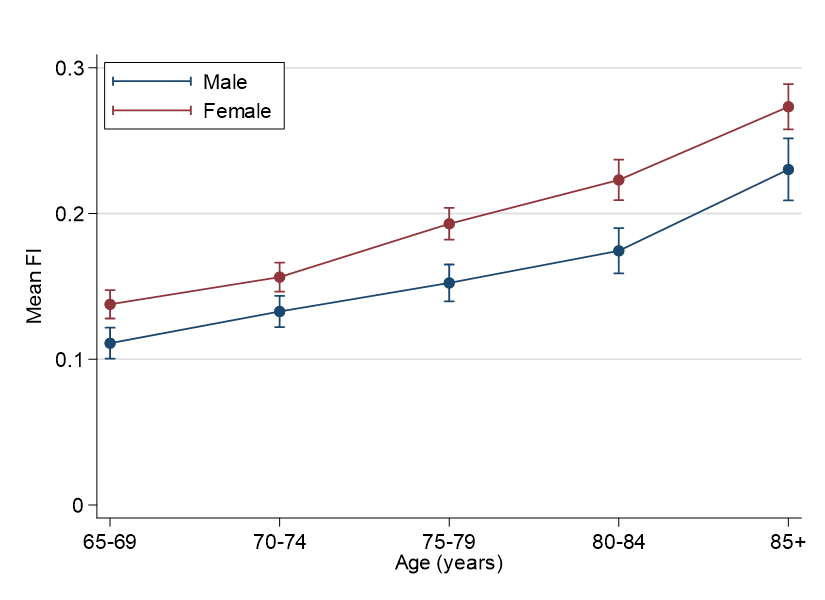


**Figure 2** Graph of mean Frailty Index (FI) with 95% confidence intervals for each sex and age group.


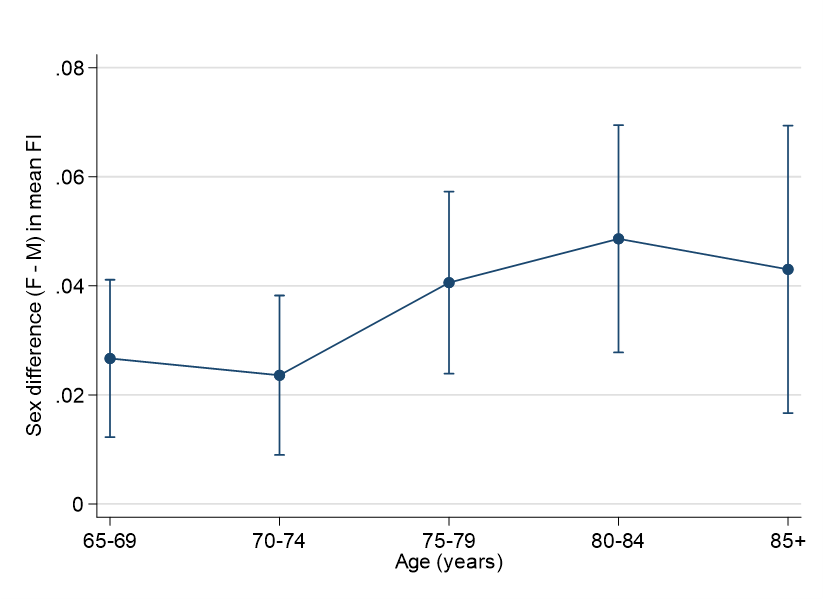


**Figure 3** Graph of sex differences in mean Frailty Index (FI) with 95% confidence intervals for each age group.

Using transformed data, the geometric mean FI was higher in females than males in all age groups (Figure 4). Similar results for main effects were detected (age: F(4,3528)=105.06, p<0.001; sex: F(1,3528)=77.29, p<0.001). The *relative* sex difference in FI was 18% (95% CI = 14 – 22, p<0.001) (Figure 5). The interaction between age and sex was not significant (F(1,3527)=0.29, p=0.587).


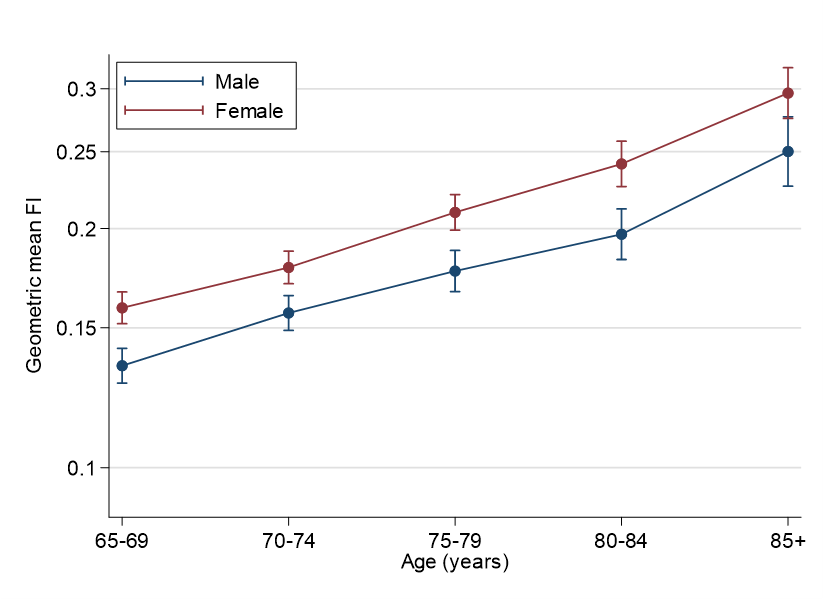


**Figure 4** Graph of geometric mean Frailty Index (FI) with 95% confidence intervals for each sex and age group.

**
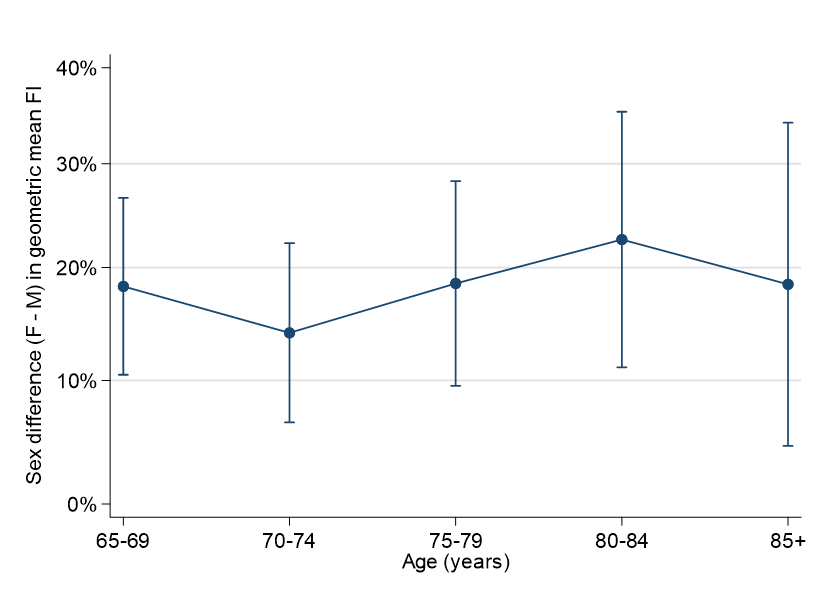
**

**Figure 5** Graph of sex differences in geometric mean Frailty Index (FI) with 95% confidence intervals for each age group.

**Derivation of the Frailty Index**

| **Domain** | **Variable** | **Value Labels** | **ELSA score** | **FI score** | **FI denominator (running total)** |
| --- | --- | --- | --- | --- | --- |
| Self-rated health | How is your health in general? | 1 very good/good  2 fair  3 bad/very bad | 1  2  3 | 0  0.5  1 | 1 |
| Mobility and Transfers | Do you have difficulty climbing one flight of stairs without resting? | 1 yes  0 no | 1  0 | 1  0 | 2 |
|  | Do you have difficulty getting in and out of bed? | 1 yes  0 no | 1  0 | 1  0 | 3 |
|  | Do you have difficulty using the toilet, including getting up or down? | 1 yes  0 no | 1  0 | 1  0 | 4 |
|  | Without using any special equipment, how much difficulty do you have walking a quarter of a mile? | 1 no difficulty  2 some difficulty  3 much difficulty  4 unable to do it | 1  2-4 | 0  1 | 5 |
|  | Do you have difficulty stooping, kneeling or crouching? | 1 yes  0 no | 1  0 | 1  0 | 6 |
|  | Do you have difficulty lifting or carrying weights over 10 pounds? | 1 yes  0 no | 1  0 | 1  0 | 7 |
| Sensory | How is your eyesight (using corrective lenses as usual)? | 1 excellent  2 very good  3 good  4 fair  5 poor  6 legally blind | 1-3  4  5-6 | 0  0.5  1 | 8 |
|  | How is your hearing (using hearing aids as usual)? | 1 excellent  2 very good  3 good  4 fair  5 poor | 1-3  4  5 | 0  0.5  1 | 9 |
| Symptoms | Are you often troubled with pain? | 1 yes  2 no | 1  2 | 1  0 | 10 |
|  | Have you fallen down in the last two years (for any reason)? | 1 yes  2 no | 1  2 | 1  0 | 11 |
|  | Have you ever fractured your hip? | 1 yes  2 no | 1  2 | 1  0 | 12 |
|  | Have you ever had any joint replacements? | 1 yes  2 no | 1  2 | 1  0 | 13 |
| Continence | In the last 12 months, have you last any amount of urine beyond your control? | 1 yes  2 no | 1  2 | 1  0 | 14 |
| pADLs | Do you have difficulty dressing (including putting on shoes and socks)? | 1 yes  0 no | 1  0 | 1  0 | 15 |
|  | Do you have difficulty bathing or showering? | 1 yes  0 no | 1  0 | 1  0 | 16 |
|  | Do you have difficulty eating, such as cutting up food? | 1 yes  0 no | 1  0 | 1  0 | 17 |
| iADLs | Do you have difficulty preparing a hot meal? | 1 yes  0 no | 1  0 | 1  0 | 18 |
|  | Do you have difficulty shopping for groceries? | 1 yes  0 no | 1  0 | 1  0 | 19 |
|  | Do you have difficulty doing work around the house and garden? | 1 yes  0 no | 1  0 | 1  0 | 20 |
|  | Do you have difficulty managing money, e.g., paying bills, keeping track of expenses? | 1 yes  0 no | 1  0 | 1  0 | 21 |
|  | Do you have difficulty taking medications? | 1 yes  0 no | 1  0 | 1  0 | 22 |
|  | Do you have difficulty making telephone calls? | 1 yes  0 no | 1  0 | 1  0 | 23 |
| Medical Conditions | Has the doctor ever told you that you have:  Hypertension | 1 yes  0 no | 1  0 | 1  0 | 24 |
|  | Angina | 1 yes  0 no | 1  0 | 1  0 | 25 |
|  | Heart Attack | 1 yes  0 no | 1  0 | 1  0 | 26 |
|  | Congestive heart failure | 1 yes  0 no | 1  0 | 1  0 | 27 |
|  | Heart murmur | 1 yes  0 no | 1  0 | 1  0 | 28 |
|  | Arrhythmia | 1 yes  0 no | 1  0 | 1  0 | 29 |
|  | Diabetes | 1 yes  0 no | 1  0 | 1  0 | 30 |
|  | Stroke | 1 yes  0 no | 1  0 | 1  0 | 31 |
|  | Chronic lung disease e.g., chronic bronchitis or emphysema | 1 yes  0 no | 1  0 | 1  0 | 32 |
|  | Arthritis including OA or rheumatism | 1 yes  0 no | 1  0 | 1  0 | 33 |
|  | Osteoporosis | 1 yes  0 no | 1  0 | 1  0 | 34 |
|  | Cancer or a malignant tumour (not minor skin cancers) | 1 yes  0 no | 1  0 | 1  0 | 35 |
|  | Cataracts | 1 yes  0 no | 1  0 | 1  0 | 36 |
| Cognition | Index of memory function:  - Today’s date  - Delayed recall – instruction  - Immediate and delayed recall – word list | 0-24 | > 1^st^ quartile  ≤ 1^st^ quartile | 0  1 | 37 |
|  | How would you rate your memory at the present time? | 1 excellent  2 very good  3 good  4 fair  5 poor | 1-3  4  5 | 0  0.5  1 | 38 |
| Mood | Has the doctor ever told you that you have:  Nervous, emotional or psychiatric problems? | 1 yes  0 no | 1  0 | 1  0 | 39 |
|  | Cesd8 score ≥ 4 | 1 yes  0 no | 1  0 | 1  0 | 40 |
